# A shifting terrain: Understanding the perspectives of walk-in physicians on their roles amid worsening primary care access in Ontario, Canada

**DOI:** 10.1371/journal.pone.0303107

**Published:** 2024-05-15

**Authors:** Braeden A. Terpou, Lauren Lapointe-Shaw, Ruoxi Wang, Danielle Martin, Mina Tadrous, Sacha Bhatia, Jennifer Shuldiner, Simon Berthelot, Niels Thakkar, Kerry McBrien, Christine Salahub, Tara Kiran, Noah Ivers, Laura Desveaux

**Affiliations:** 1 Institute for Better Health, Trillium Health Partners, Mississauga, Ontario, Canada; 2 Division of General Internal Medicine and Geriatrics, University Health Network and Sinai Health System, Toronto, Ontario, Canada; 3 Institute of Health Policy, Management and Evaluation, University of Toronto, Toronto, Ontario, Canada; 4 Department of Medicine, University of Toronto, Toronto, Ontario, Canada; 5 Women’s College Institute for Health System Solutions and Virtual Care, Women’s College Hospital, Toronto, Ontario, Canada; 6 Institute for Clinical Evaluative Sciences, Toronto, Ontario, Canada; 7 Department of Family and Community Medicine, University of Toronto, Toronto, Ontario, Canada; 8 Department of Family Medicine, Women’s College Hospital, Toronto, Ontario, Canada; 9 Women’s College Research Institute, Women’s College Hospital, Toronto, Ontario, Canada; 10 Leslie Dan Faculty of Pharmacy, University of Toronto, Toronto, Ontario, Canada; 11 Peter Munk Cardiac Centre, University Health Network, Toronto, Ontario, Canada; 12 Département de Médecine de Famille et de Médecine D’urgence, Université Laval, Laval, Quebec, Canada; 13 College of Nurses of Ontario, Toronto, Ontario, Canada; 14 Department of Family Medicine, University of Calgary, Calgary, Alberta, Canada; 15 Department of Community Health Sciences, University of Calgary, Calgary, Alberta, Canada; 16 Supports, Systems and Outcomes Department, University Health Network, Toronto, Ontario, Canada; 17 Department of Family and Community Medicine, St. Michael’s Hospital, Toronto, Ontario, Canada; Tabriz University of Medical Sciences, IR Iran, ISLAMIC REPUBLIC OF IRAN

## Abstract

**Background:**

High-quality primary care is associated with better health outcomes and more efficient and equitable health system performance. However, the rate of primary care attachment is falling, and timely access to primary care is worsening, driving many patients to use walk-in clinics for their comprehensive primary care needs. This study sought to explore the experiences and perceived roles and responsibilities of walk-in physicians in this current climate. Methods: Qualitative interviews were conducted with nineteen physicians currently providing walk-in care in Ontario, Canada between May and December 2022.

**Results:**

Limited capacity for continuity and comprehensiveness of care were identified as major sources of professional tension for walk-in physicians. Divergent perspectives on their roles were anchored in how physicians viewed their professional identity. Some saw providing continuous and comprehensive care as an infringement on their professional role; others saw their professional role as more flexible and responsive to population needs. Regardless of their professional identity, participants reported feeling ill-equipped to manage the swell of unattached patients, citing a lack of time, resources, connectivity to the system, and remuneration flexibility. Conclusions: As practice demands of walk-in clinics change, an evolution in the professional roles and responsibilities of walk-in physicians follows. However, the resources, structure, and incentives of walk-in care have not evolved to reflect this, leaving physicians to set their own professional boundaries with patients. This results in increasing variations in care and confusion across the primary care sector around who is responsible for what, when, and how.

## Introduction

A strong foundation of primary care is crucial to achieving better health for all [1]. Patients with access to primary care generally have better health outcomes, owing to more preventative care, earlier treatment for potentially serious issues, and better management of chronic diseases [2–8]. Unfortunately, access to primary care is declining compared to previous years, with approximately 6.5 million Canadians (17%) lacking a regular attachment to a primary care clinician [9, 10]. In Ontario, Canada’s most populous province, the number of unattached patients has grown from 1.8 to 2.2 million in the past two years, disproportionately affecting newcomers to Ontario and those living in the poorest and most racialized neighborhoods [11]. Ontarians who experience access gaps are increasingly relying on walk-in clinics as a substitute [12, 13], raising concerns as to whether and how their primary care needs are being met [14–16].

Walk-in clinics are a popular alternative due to their extended hours, shorter wait times, and ease of access [7, 14–16]. Despite their popularity, walk-in clinics are not intended for continuity of care nor for comprehensive care provision [7, 16]. We distinguish independent walk-in clinics that provide episodic care from urgent care centres that provide services akin to an emergency department and from after-hours clinics affiliated with relationship-based primary care teams [14, 16]. Walk-in clinics prioritize high-volume, episodic care, an approach that is not well-aligned with managing an increasingly complex and multi-morbid primary care population [7, 14–18]. Accordingly, patients with chronic diseases and other complex presentations may have health needs that fall outside the typical scope of walk-in care, presenting challenges for both patients and physicians [14–16, 19–21]. In addition, this raises equity concerns as walk-in patients are disproportionately from underserved communities that would benefit from social determinants of health-oriented and relationship-based models of care [15, 16, 19].

With the number of unattached patients rising [10, 11, 13] and timely access to primary care worsening [9, 11], there is an increasing demand for longitudinal and comprehensive care provision in walk-in clinics [19, 20, 22]. Despite this, little is currently known about the experiences and perceived roles and responsibilities of walk-in physicians, with most studies relying on data from the 20-year-old *Ontario Walk-In Clinic Study* [16, 23, 24]. In this study, most walk-in physicians saw preventative care and continuity of care as neither their focus nor their responsibility [16, 24]. In light of growing pressures, understanding whether and how the attitudes of walk-in physicians have changed in recent years is central to identifying a path forward at a system level.

We conducted interviews with Ontario walk-in physicians to better understand their experiences, perceived roles and responsibilities, and practice-related challenges. Through this work, we hope to better understand how the walk-in care model can complement the primary care and acute care systems, with the ultimate goal of informing and optimizing care delivery within Ontario, Canada.

## Materials and methods

### Study design

This qualitative study explored the experiences and perceived roles and responsibilities of walk-in physicians. We used semi-structured interviews to gather data from physicians actively practicing in walk-in clinics in Ontario, Canada. This study was nested within a larger program of work undertaken to understand whether and how care models can be improved to better meet the care needs of individuals in Ontario. This study was reviewed and approved by Trillium Health Partners’ Research Ethics Board (ID #1091), and all physicians provided informed consent for their involvement. Data collection took place between May and December 2022.

Tong, Sainsbury, and Craig’s Consolidated Criteria for Reporting Qualitative Studies (COREQ) [25], a 32-item checklist, was used to ensure explicit and comprehensive reporting of qualitative study procedures.

### Setting

In Ontario, the majority of the population (83%) has a primary care clinician (or a family medicine specialist) as their first point of contact within the healthcare system [26], with approximately 14,500 physicians providing primary care services as of 2021 [9, 11, 26]. However, not all these primary care clinicians provide relationship-based primary care, and the proportion that do is shrinking. Many physicians left the profession during COVID-19, with approximately 3% of primary care clinicians leaving relationship-based practice between March and September of 2020 alone [9].

Medically necessary visits to primary care clinicians in Ontario are covered by the provincial health insurance plan without deductible or co-pay. Primary care clinicians may work in a variety of practice settings, such as solo practices, group practices, or Community Health Centres, and have different remuneration schedules available to them depending on the practice setting, ranging from purely fee-for-service to capitation to salary-based. Most primary care clinicians in Ontario are now paid through a blended capitation model. Under this model, physicians receive a fixed amount of money per patient on their roster based on factors such as age, sex, and health status (similar to capitation) as well as additional payments for specific services performed (similar to fee-for-service) [27, 28]. Capitation models may also incorporate pay-for-performance bonuses, shared savings, and bundled payments. Some of these bonuses may be negated if a group’s rostered patient accesses care outside of their primary care home [27]. Henceforth, we use the term “primary care” to refer to primary care models providing relationship-based care and remunerated using a capitation or blended capitation model.

In addition to primary care practices, many physicians trained in family and/or general medicine also work part-time or full-time in walk-in clinics. When working in walk-in clinics, physicians primarily provide episodic care and are remunerated fee-for-service [17, 18]. Unlike capitation, the fee-for-service model does not include pay-for-performance bonuses.

Traditionally, both primary care and walk-in care were predominantly delivered in-person. However, in response to the COVID-19 pandemic, Ontario implemented various reforms to billing codes and reimbursement mechanisms to support the delivery of virtual care. Because of these reforms, virtual walk-in clinics proliferated, becoming a popular avenue for care delivery in Ontario today [19]. They offer direct-to-consumer consultations with physicians through video, phone, or text-based platforms, providing a convenient option for patients seeking medical advice and treatment remotely, and are remunerated on a fee-for-service basis.

Ontario does not have a province-wide electronic health record system, which means that a patient’s medical history is managed independently by a range of health information custodians, resulting in fragmented information that is not necessarily accessible by physicians working in walk-in clinics.

### Recruitment

A purposive sampling strategy was used to recruit physicians for this study, focusing on those whose primary practice setting was an in-person or virtual walk-in clinic in Ontario. According to a 2019 Physicians and Surgeons of Ontario survey, 1,103 physicians reported that a “walk-in clinic or episodic care clinic outside of a hospital” was their primary practice setting.

Three parallel channels were used to recruit participants. First, a list of clinics providing walk-in care (including both in-person and virtual) was developed based on the Canadian Physician Database group billing numbers and business names from the Ministry of Health [19, 29]. A *Recruitment Letter* was distributed by fax or email to all 472 clinics asking for their support in circulating an *Invitation Letter* and *Letter of Information* to physicians practicing in their clinics. Second, the *Invitation Letter* and *Letter of Information* were shared within the research team members’ networks via email. Lastly, brief study details and an invitation to participate was circulated by research team members on social media, including Twitter, LinkedIn, and Facebook.

Interested participants were asked to provide demographic information, which was used to purposively recruit a sample with balanced representation across gender and years in practice. No participants expressing interest in the study refused to participant after scheduling interviews.

### Data collection

A semi-structured interview guide (S1 Text) was developed based on the Theoretical Domains Framework (TDF) [30] and the Consolidated Framework for Implementation Research (CFIR) [31]. The interview guide asked participants to describe their experiences providing in-person and/or virtual walk-in care and to compare these experiences with that of providing primary care, where applicable. Some of these questions focused on clinic-level factors, such as patient characteristics, available resources, and workloads, while others focused on system-level factors, such as remuneration schedules, external policies, and incentives. Other questions probed individual-level reasons for practicing walk-in care and individual-level perspectives on what constitutes good vs. bad walk-in care.

The interview guide was piloted with physicians trained in family medicine to ensure clarity and comprehensive attention to individual-, clinic-, and system-level factors. Interviews were conducted by a Research Associate (RW, PhD, female) with extensive experience with health system research. RW has previous experience with semi-structured interviewing and was trained by the Scientific Director of THP’s Institute for Better Health. The interviewer had no prior relationship with participants.

Interviews were conducted virtually on Zoom. Audio recordings were sent to an independent third party for transcription and de-identification.

### Data analysis

Transcripts were independently coded in MAXQDA by two coders (RW, BT). Data analysis was performed using a deductive and inductive coding style that followed the principles of qualitative description for a content analysis [32, 33]. Codes were first deductively mapped to corresponding TDF and CFIR constructs. Inductive coding was applied when themes emerged that did not fit within the definitions of the pre-defined codes. Codes were reviewed to ensure consistency and clarity, and discussions were held on points of disagreement. Where consensus was not achieved, a third team member (LD) was brought in to discuss and resolve. Data collection and analysis was an iterative process, with the interview guide and coding framework evolving in response to emerging insights. Thematic saturation was reached between 15–17 interviews and an additional 2–4 interviews were conducted to confirm no additional insights were offered [34] and to meet the recruitment goal of having a balanced sample of early- and late-career walk-in physicians.

## Results

A total of 19 interviews were conducted, ranging from 33 minutes to 1 hour and 7 minutes in duration (average duration = 54 minutes). Participant characteristics can be found in Table 1. The results highlight four key themes of walk-in care and identify how individual attitudes, beliefs, and the overarching context influence care provision in walk-in clinics.

**Table 1:** Characteristics and demographics of walk-in physician participants.

Demographics	Participant (N = 19)
**Gender**	
** Male**	7 (37%)
** Female**	12 (63%)
**Age**	
** Median [Min, Max]**	35 [29, 57]
**Medical Training**	
** Canada**	16 (84%)
** Outside Canada**	3 (16%)
**Years after Graduating**	
** 0–5 years**	9 (47%)
** 6–10 years**	6 (32%)
** 11–20 years**	4 (21%)
**Community Size**	
** Large urban**	18 (95%)
** Small or medium population**	1 (5%)
**Percentage of Patient Population Estimated to be of Recent Immigrant Status**	
** Median [Min, Max]**	45.5 [5, 86]
**Average Number of Primary Care Hours per Week**	
** 0–9 hours**	6 (32%)
** 10–19 hours**	3 (16%)
** 20–29 hours**	8 (42%)
** 30–39 hours**	2 (10%)
**Average Number of In-Person Walk-In Care Hours per Week**	
** 0–9 hours**	13 (68%)
** 10–19 hours**	3 (16%)
** 20–29 hours**	1 (5%)
** 30–39 hours**	2 (10%)
**Average Number of Virtual Walk-In Care Hours per Week**	
** 0–9 hours**	11 (58%)
** 10–19 hours**	6 (32%)
** 20–29 hours**	1 (5%)
** 30–39 hours**	1 (5%)

### What drives a physician to practice walk-in care?

The most common reason for practicing walk-in care was the flexibility and convenience it offered in accommodating physicians’ lifestyles. This was cited predominantly by physicians working in virtual walk-in clinics, although one physician practicing in-person walk-in care saw not having the responsibility of a full roster as another form of convenience. Having children was another lifestyle factor that made the flexibility of the walk-in care model favourable for several male and female participants. Three of these participants started practicing on virtual care platforms due to the pandemic and continued to do so after loosening restrictions because of the convenience.

*“It’s more of a personal time of life right now for me*. *Because I have two kids who have been at home with me full-time and so*, *I do not have the capacity to open up a practice personally*. *At this moment*, *I don’t want to bring any work home*, *and I like the flexibility of choosing my shifts depending on when childcare is available*.” *P5 [Late Career]*

Other participants described a preference for episodic care, citing a lack of interest in “following patients over many years” and a preference for “short and sweet” [P7] care provision.

Three participants, all early in their careers, saw walk-in clinics as an effective place to practice family medicine while working to fill their primary care rosters. In particular, practicing walk-in care was seen as “supplementing inefficiencies in [their] schedules” [P11] and, for one participant, a means of actually “recruiting new patients” [P15].

Most of the participants (14/19) practicing walk-in care also had their own primary care practice. Several of these participants cited the demand for walk-in care as their main driver.

*“I was mostly motivated [to practice walk-in care] because I felt really bad for what’s going on lately with the demand*. *I don’t need that job by any means*. *It’s not my main job*. *It’s only half a day to a day a week*. *But I felt like there was a lot of patients that I was seeing virtually who needed in-person care and there’s not a lot of in-person care available*.” *P11 [Late Career]*

Several participants referenced difficulties finding in-person work as a driver, especially among recent medical graduates, who cited the temporary reduction in the availability of primary care locums caused by COVID-19 as a pressure. This lack of availability was replaced by the proliferation of virtual care services.

*“Ever since I graduated*, *I’ve been doing locums for other doctors*. *And when COVID started*, *there wasn’t much locum availability for a period of time*, *because nobody was travelling*. *Everything was just a mess*. *Then a colleague of mine told me about Rocket Doctor*, *and I thought*, *OK*, *that’s something to do while I look for my next locum*. *Initially I was working on it quite a bit*, *but for a long time*, *even when I did find interesting locums*, *I was putting in a couple of hours a week*, *every week*, *into it*.” *P16 [Early Career]*

In addition to lifestyle and preference factors, some participants cited the fee-for-service model was an initial driver to practice walk-in care. The fee-for-service model was seen as an effective remuneration schedule for “boosting [their] income” [P17]. One physician described the model as being especially lucrative in walk-in clinics compared to other fee-for-service settings.

### What are the perceived roles and responsibilities of walk-in physicians?

Participants unanimously saw their primary role as filling gaps within the healthcare system. Specifically, they saw themselves as bridging access to primary care to “prevent [patients] from going to the emergency room” [P12], which was perceived as a benefit to both the patient and to the healthcare system. To achieve this goal, walk-in physicians commonly described triaging patients and minimizing system costs as central to their professional role.

*“I provide care for a very focused issue*, *and I let [the patient] know what other issues need to be looked at and what the urgency of those are*. *So*, *whether that’s something that can wait a week*, *a month*, *five years*, *ten years*, *I try to explain that to them […]*. *Oftentimes*, *there’s a misalignment between what a patient really thinks they need and what the big problem is*. *So*, *I try to identify the problems and then I try to explain how to triage those problems and where to access the right care for that*.” *P11 [Late Career]*

In addition to triaging non-urgent issues, participants saw themselves as serving as a “stopgap” for attached patients experiencing difficulties with timely access, which was perceived as an increasingly prevalent issue among walk-in patients. This need was described as inevitable since “family physicians simply cannot be there for patients twenty-four seven” [P16], with participants unanimously expressing a perceived need for both primary and walk-in care settings. When serving as a stopgap, walk-in physicians flagged the importance of sending the patient back to their primary care clinician after the walk-in appointment to prevent further disruptions to care continuity.

Walk-in physicians saw themselves as providing a specific style of care characterized by quick visits with patients to address one-off, acute care needs. This style of care was largely seen as being dictated by the fee-for-service model. The fee-for-service model was also seen as influencing the nature of the patient-physician interaction to be more transactional rather than relational. This was viewed as a benefit to the physician by way of increasing compensation and allowing them to focus on what they perceived as their immediate goal of keeping patients out of the emergency department.

*“In a walk-in setting that’s almost how you have to practice*. *[Spending] 20 more minutes establishing rapport with a patient may be important if they are your rostered patient*. *But it doesn’t make sense in a fee-for-service setting to do that*, *right*? *It’s very much like what is my patient’s current medical need*, *how can I bridge the gap and stop them from going to the ER*?” *P18 [Early Career]*

### What current practice-related pressures are experienced by walk-in physicians?

Many participants highlighted the fee-for-service model as contributing to the current pressures in walk-in clinics. Though this model was acknowledged for effectively compensating episodic care, it was described as a deterrent to providing longitudinal and comprehensive care, thereby reinforcing the transactional nature of walk-in clinics.

*“The only issue is*, *as walk-in doctors*, *you’re not incentivized to do [preventative care]*, *because in a primary care practice*, *you actually get paid bonuses for providing preventative care*. *But in a walk-in*, *you don’t*. *It’s all fee-for-service*, *or you can’t bill for those preventative care screening codes and things like that*, *right*? *So*, *I think that may be what disincentivizes (sic) walk-in clinics from doing that*.” *P9 [Early Career]*

Most walk-in physicians saw the fee-for-service model as needing to evolve to better reflect the growing demands for longitudinal and comprehensive care in walk-in clinics. Some physicians were in favour of an hourly-, or salary-based model, while others advocated for “more nuanced ways of remuneration” [P19], such as having “modifiers for complexity and age built into the billing structure” [P10]. This was seen as having the benefit of encouraging physicians to take the time necessary to deliver high-quality care for the more complex patients.

Participants emphasized other obstacles to providing longitudinal and comprehensive care, including the variable schedules of walk-in physicians. This complicates follow-up and often contradicts the prevailing system expectation that the ordering physician is responsible for follow-up. Several participants stated that follow-up often falls to the primary care clinician. However, this is dependent on whether the primary care clinician receives information about the visit, either through the patient’s electronic health record, contact from the walk-in physician, or communication from the patient. Among these communication pathways, the latter was perceived to be most common. A few participants acknowledged this lack of consistency as concerning from a patient outcome perspective, as it leads to a lack of continuity in patient care at best and patients sometimes “getting lost to follow-up” [P13] at worst. To address this, a few participants mentioned adopting a team-based approach to managing follow-up, emphasizing charting and note sharing among physicians within the clinic. While this creates value for patients who frequently access the clinic, concerns were expressed regarding the effectiveness of this approach.

*“So*, *you end up having*, *let’s say*, *10 different physicians managing a patient over the course of a year for one or two health conditions*. *But all of those physicians might have slightly different variations in terms of how they would manage that chronic condition*. *And in that situation*, *I really don’t think a patient gets the best care*, *certainly when compared to having their own designated primary care provider*.” *P10 [Late Career]*

The majority of participants cited the lack of access to centralized patient records as another limitation. While some participants felt a sense of responsibility for managing their patients’ care needs despite limited information, others viewed it as outside their scope for this reason.

*“A walk-in clinic doctor isn’t obliged to store and coordinate your paperwork from every specialist you see*. *They don’t really take true ownership of you*, *in a sense*. *They’re not responsible for saying*, *‘Hey*, *I know you’re here for your earache*, *but I see here that we haven’t done a pap smear for five years*.*’ They don’t keep those records*, *they don’t think along those lines*, *because they’re not supposed to*.” *P16 [Early Career]*

In addition to the challenges at the practice level, participants identified a lack of clear, system-level standards as contributing to the difficulties of delivering walk-in care. This issue was perceived as leading to the individual creation of and variability in standards of care at the clinic level.

### How do physicians perceive their professional role amid growing demands for longitudinal and comprehensive care?

Walk-in physicians had divergent perspectives on providing longitudinal and comprehensive care in walk-in clinics based on their professional identity. Some physicians perceived their professional identity as adaptable and influenced by the overall care needs of their patients, and therefore considered longitudinal and comprehensive care to be within their scope. While recognizing the challenges associated with such adjustments, they acknowledged current patient needs as determining their roles and responsibilities.

*“If the patient is unattached and they need medical care*, *the role of that walk-in care can be completely comprehensive*. *And that’s oftentimes what ends up happening with some patients that like*, *have diabetes*, *and don’t have anywhere to go*. *They have to go somewhere every three months*. *So*, *if they can go to the same walk-in clinic and get semi- or partially continuous care*, *it’s better than nothing*. *The doctor that works there should be more than capable to do all those types of services*, *even though maybe they don’t want to*.” *P2 [Early Career]*

In contrast, certain physicians viewed their professional identity as determined by their practice setting. For these physicians, their professional scope was firmly established, and they held a more rigid perception of their role, which limited their willingness to engage in longitudinal and comprehensive care provision.

*“The challenge is when you have patients who don’t have any access to primary care*, *or who have challenges with their primary care physician–and then who actually force it upon the physicians or the clinics that provide this episodic care–to adapt and be a bit more of a longitudinal type of care provider*, *but it becomes very difficult*.” *P10 [Late Career]*

Amid these divergent perspectives, walk-in physicians found themselves in the position of having to navigate their own approach, including establishing boundaries with patients on a case-by-case basis, resulting in undue stress.

*“I think my biggest concern with patients has always been the ones that don’t have family doctors*. *Because I’m always like*, *‘Where is this relationship going*?*’ I don’t want to cut somebody off who doesn’t have a family physician; that doesn’t feel good*. *But I can’t take care of them the way that they need to be taken care of and be a walk-in physician That’s probably the hardest aspect of it*, *to know where to draw that boundary between a walk-in physician and a family physician*. *You have to*, *because if you don’t*, *then you become their family physician*. *But that doesn’t feel good*, *because everyone needs a family physician*, *everybody needs someone taking care of their big picture*.” *P16 [Early Career]*

## Discussion

This study explored the perspectives of walk-in physicians as they navigate a shifting climate of professional demands. The growing number of unattached patients and their primary care needs were perceived as a tension among physicians as they balance the transactional nature of walk-in clinics with the increasing demand for longitudinal and comprehensive care. Participants expressed divergent perspectives on how this tension intersected with their professional identity and their resultant approach to care. This change in population needs was also seen as exacerbating variations in the nature and quality of walk-in care. In the following paragraphs, we contextualize this tension, its perceived drivers, and highlight some evidence-based opportunities for evolution of the walk-in care model.

### Drivers

Participants described the tension as stemming from a lack of role clarity and a disconnect between the intended nature of walk-in care and the current demands of their practice. Role ambiguity was exacerbated by varying perceptions of professional identity and a lack of a clearly defined scope for walk-in clinics, resulting in role conflict and stress [35–37]. In addition to being a source of conflict [36,37], role ambiguity allows for varied expectations of physicians to form based on their priorities. The fee-for-service model incentivizes high-volume, transactional services [38–42], likely at the expense of more comprehensive care provision [4–6]. Accordingly, walk-in physicians seeking to maximize remuneration can structure their practice to prioritize volume, for example, by limiting patients to one problem per visit regardless of need or complexity [17,18]. Without the performance bonuses of capitation-based models [27,28], the fee-for-service model does not incentivize preventative care or chronic disease management for walk-in patients. Despite having a greater frequency of visits, Lofters et al. [43] found unattached patients visiting walk-in clinics to have lower rates of cancer screening compared to patients with a primary care clinician. This aligns with previous findings of uncertainty around the place for screening in walk-in clinics [16,24].

The fee-for-service model was not the sole driver of care provision, as participants highlighted the structure of walk-in clinics, available resources, and system connectivity as compounding the problem. For example, the responsibility for follow-up in Ontario typically lies with the ordering physician or their designated care team [7,8,14]. However, some participants mentioned ordering upwards of 10–15 tests per day, making it difficult to follow up on all of them in light of inconsistent work hours. Moreover, the fee-for-service model does not remunerate follow-up phone calls if initiated by the physician [17,18]. Consequently, most walk-in physicians encourage patients to seek follow-up by returning to the walk-in clinic, but such visits are likely to be with another physician who may have a different perspective on clinical management. This disrupts continuity and places follow-up responsibility on the patient, likely exacerbating inequities for a population of patients that are largely from underserved communities and experience difficulties navigating the healthcare system [15,19]. Moreover, this disincentive may increase healthcare system costs by encouraging repeated physician visits for the same issue [14–16,19,20,44].

Participants also identified access (or lack thereof) to a centralized, electronic health record (EHR) as another limitation. This limitation is particularly evident in providing care for the nearly 23% of unattached patients with chronic diseases who require preventative care and longitudinal management [14–16]. Access to complete medical histories, tests results, and imaging reports is not only crucial for effective care coordination, but prevents duplication of services, ultimately reducing healthcare system costs [45–48]. The implementation of a system-wide EHR can improve care coordination, reduce costs, and provide patients with access to their own health information [48–50]. Moreover, owing to standardized reporting and centralized storage, a system-wide EHR enables better evaluation of health trends and outcomes [50,51]. Conversely, Ontario’s lack of a system-wide EHR hampers care delivery and coordination between walk-in clinics and primary care settings [45,52,53].

### System-level reforms

In the years ahead, many clinicians practicing relationship-based primary care are poised to retire in Ontario [9,11], likely exacerbating the primary care access problem. This concern is compounded by a perceived decline in the interest in family medicine in general, with fewer medical graduates choosing comprehensive practice in favour of more focused practice [9,19,54,55]. In the absence of aligned incentives, there is a risk that physicians will continually be drawn to walk-in care due to its potential for higher compensation and perceived flexibility [14,16,24]. Flexibility was the most oft-cited driver to practice walk-in care, highlighting the need for policies to alleviate the administrative burdens and introduce more flexibility in primary care settings [26,56,57]. Increasing funding for inter-professional and team-based care models, along with payment reforms, may improve population-based access to primary care [57–59]. This, paired with regionally organized after-hours clinics, could help expand timely access to care, reducing the need for patients to seek care outside their designated primary care home. By re-aligning incentives, more physicians may choose to practice relationship-based primary care, resulting in bolstered capacity and access that will deliver better population outcomes [58,59].

Establishing temporary pathways for shared care, communication, and referrals between walk-in and primary care settings could facilitate seamless care transitions and coordinated follow-up, even in the absence of primary care attachment. This could involve nesting walk-in clinics within enrolling primary care practices, allowing for shared accountability, alignment on funding, and formal affiliations based on geographic and population boundaries [60,61]. This approach has been effective in the United States with psychiatric services. By partnering with psychiatric walk-in clinics, same-day access for psychiatric care is provided to patients who frequently miss appointments or prefer ambulatory-style care delivery, while enrolling psychiatrists benefit by receiving referrals [61]. Primary care funding structures could also be extended to walk-in physicians to provide them with explicit preventative care incentives or bonuses for enrolling unattached patients [40–42,60–62]. However, it is important to underscore that these reforms would best serve as interim solutions while striving towards the long-term objective of achieving relationship-based primary care for all [1,63,64].

### Limitations

These findings reflect a sample of physicians in Ontario trained in family and/or general medicine. As participation was voluntary and recruitment was largely electronic, our sample may be subject to selection bias, whereby walk-in physicians who were not satisfied with some aspect of their practice experience or who valued the walk-in care model may have been more inclined to participate. In addition, these findings are based on self-report and would benefit from triangulation with objective measures of availability, resources, and funding structures and their impact on care provision. While we purposively recruited a sample with a balanced representation of early- and late-career physicians, we were only able to recruit one physician practicing in a rural setting. This may reflect the reality that walk-in clinics are primarily located in larger urban areas [29]; however, future work should explore whether and how practice experiences and perspectives differ between rural and urban settings.

## Conclusions

Walk-in clinics have been surrounded with controversy since their inception [16,23,24], with many of the challenges discussed here echoing findings from two decades ago by Brown et al. [16]. Ontario’s evolving population demographics have intensified these issues, driven by a surge in population and the increasing proportion of new immigrants into the province [65], resulting in a substantial rise in unattached patients. While walk-in clinics serve as a temporary solution for many of these patients’ healthcare needs, they are ill-equipped and under-resourced to provide longitudinal and comprehensive care. Consequently, walk-in physicians face the dilemma of defining their professional boundaries amidst shifting patient demands. This dynamic is likely to exacerbate variations in care delivery within walk-in settings, emphasizing the urgent need for policies and standards to clarify the role of walk-in clinics within Ontario’s healthcare framework.

## Supporting information

S1 Text Interview guide. (DOCX)
