# Supplementary material for: A shifting terrain: Understanding the perspectives of walk-in physicians on their roles amid worsening primary care access in Ontario, Canada
Source: PLoS One. 2024 May 15;19(5):e0303107. doi: 10.1371/journal.pone.0303107 (PMC11095764; doi:10.1371/journal.pone.0303107)
Supplement: S1 Text — (DOCX) [file pone.0303107.s001.docx]

**Interview Guide**

**Preamble**

The purpose of this interview is to explore your experiences providing walk-in care, perceptions around variations in clinical decision-making between walk-in care and comprehensive primary care, and your suggestions on strategies to facilitate quality improvement. The interview should take approximately 45-60 minutes. If any question makes you feel uncomfortable, you can skip that question. You may stop participating at any time.

*For those who consented to audio recording:*

With your permission, I would like to audio record the interview because I don’t want to miss any of your comments. May I turn on the audio recorder?

*For those who declined audio recording:*

As per your requirement, I won’t audio record the interview. My colleague will help take notes today.

**Experiences and Perceptions of Providing Walk-In Care**

1. I would like to start with your general experiences providing walk-in care in Ontario. Can you describe the walk-in clinic you work in? (e.g., Who owns it? How many physicians work there? What are the type of patients you see?)

*Prompt:*

Scope of medical care, characteristics of practice organization (e.g., number, set-up of primary practice organization), patient population (e.g., % of recent immigrants, % of patients with a family physician, medical needs), etc.

*Follow-up:*

1. Have you noticed any differences or patterns across patient groups (e.g., immigrant status, family physician enrolment status)?
2. How does this differ between in-person and virtual walk-in care? (if applicable)
3. What factors motivated you to provide in-person or virtual walk-in care?
4. How would you describe the roles and responsibilities of walk-in physicians?

*Prompt:*

Current gaps in the healthcare system and walk-in physicians’ contribution (e.g., access, patient preference), scope of medical care (e.g., preventive care, antibiotics prescribing)

1. Are there any tensions between the expectations placed upon walk-in physicians and your own perception of the role?

*Prompt:*

Managing patients needing preventative or chronic disease management

1. What are the things that concern you or keep you up at night?

**Comparing Care across Settings**

1. What are the differences and similarities between walk-in care and comprehensive primary care?
2. *For those who only provide walk-in care*:

From your perspective, what does it mean to be a good walk-in physician? How is this similar or different to being a good comprehensive primary care physician?

*For those who provide walk-in care and comprehensive primary care*:

I have noticed that you provide both walk-in care and comprehensive primary care. From your perspective, what does it mean to be a good physician for the walk-in work? How is this similar or different to being a good physician for the comprehensive primary care work?

*Follow-up:*

How do you define “good walk-in care”?

**Factors Influencing Clinical Behaviours**

Thank you for sharing your experiences and perceptions of providing walk-in care. I can see that [reflect back what has been shared], and sometimes it can be challenging. I’d like to shift to exploring the factors that influence your clinical decision-making in the context of walk-in care.

1. Please describe an example of when you were able to provide what you felt was good walk-in care and one where you found it challenging to provide walk-in care or needed more support.
2. You mentioned X, Y, Z. What factors influence clinical decision-making more broadly in walk-in settings?

*Prompt:*

Patient factors, influence from colleagues, available resources, billing codes, workloads, etc.

*Follow-up:*

Please give me an example of how X factor influences clinical decisions.

**Factors Influencing Differences in Quality of Care**

I’d like to shift our focus slightly to discuss patient outcomes and experience. We found some differences in [Indicator 1]/[Indicator 2]/[Indicator 3] between patients who receive walk-in care and those who receive comprehensive primary care. I am not a health professional, so I’m hoping we can have a conversation brainstorming what you see as the possible reasons behind these differences?

1. For [Indicator 1]/ [Indicator 2]/ [Indicator 3], have you noticed anything within your clinic or among your colleagues that would help explain this variation?

*Prompt:*

Patient factors, influence from colleagues, available resources, billing codes, workloads, etc.

*Follow-up:*

- 1. Please give me an example of how *X* factor influences variation.
  2. What strategies do you think would help address [insert factors mentioned]?
  3. How would you envision [insert strategy] would be implemented?

1. What, if anything, would you hope would be different for walk-in care or comprehensive primary care in the next few years?
2. Is there anything else you would like to mention we haven’t discussed today?

Those are all the questions I have for you. I appreciate the time and insights that you’ve given me today.
